# The *Lotus japonicus* ROP3 Is Involved in the Establishment of the Nitrogen-Fixing Symbiosis but Not of the Arbuscular Mycorrhizal Symbiosis

**DOI:** 10.3389/fpls.2021.696450

**Published:** 2021-11-12

**Authors:** Ivette García-Soto, Raphael Boussageon, Yareni Marlene Cruz-Farfán, Jesus Daniel Castro-Chilpa, Liz Xochiquetzal Hernández-Cerezo, Victor Bustos-Zagal, Alfonso Leija-Salas, Georgina Hernández, Martha Torres, Damien Formey, Pierre-Emmanuel Courty, Daniel Wipf, Mario Serrano, Alexandre Tromas

**Affiliations:** ^1^Centro de Ciencias Genómicas, Universidad Nacional Autónoma de México, Cuernavaca, Mexico; ^2^Programa de Doctorado en Ciencias Bioquímicas, Centro de Ciencias Genómicas, Universidad Nacional Autónoma de México, Cuernavaca, Mexico; ^3^Agroécologie, AgroSup Dijon, CNRS, Université de Bourgogne, INRAE, Université Bourgogne Franche-Comté, Dijon, France; ^4^La Cité College, Bureau de la Recherche et de l’Innovation, Ottawa, ON, Canada

**Keywords:** symbiotic nitrogen fixation, ROP, rho-GTPase, *Lotus japonicus*, arbuscular mycorrhizal symbiosis

## Abstract

Legumes form root mutualistic symbioses with some soil microbes promoting their growth, rhizobia, and arbuscular mycorrhizal fungi (AMF). A conserved set of plant proteins rules the transduction of symbiotic signals from rhizobia and AMF in a so-called common symbiotic signaling pathway (CSSP). Despite considerable efforts and advances over the past 20 years, there are still key elements to be discovered about the establishment of these root symbioses. Rhizobia and AMF root colonization are possible after a deep cell reorganization. In the interaction between the model legume *Lotus japonicus* and *Mesorhizobium loti*, this reorganization has been shown to be dependent on a SCAR/Wave-like signaling module, including Rho-GTPase (ROP in plants). Here, we studied the potential role of ROP3 in the nitrogen-fixing symbiosis (NFS) as well as in the arbuscular mycorrhizal symbiosis (AMS). We performed a detailed phenotypic study on the effects of the loss of a single ROP on the establishment of both root symbioses. Moreover, we evaluated the expression of key genes related to CSSP and to the rhizobial-specific pathway. Under our experimental conditions, *rop3* mutant showed less nodule formation at 7- and 21-days post inoculation as well as less microcolonies and a higher frequency of epidermal infection threads. However, AMF root colonization was not affected. These results suggest a role of ROP3 as a positive regulator of infection thread formation and nodulation in *L. japonicus*. In addition, CSSP gene expression was neither affected in NFS nor in AMS condition in *rop3* mutant. whereas the expression level of some genes belonging to the rhizobial-specific pathway, like *RACK1*, decreased in the NFS. In conclusion, ROP3 appears to be involved in the NFS, but is neither required for intra-radical growth of AMF nor arbuscule formation.

## Introduction

Roots of legumes interact with multiple soil beneficial microorganisms, including bacteria and fungi. Legumes can form two main root symbioses: the nitrogen-fixing symbiosis (NFS) and the arbuscular-mycorrhizal symbiosis (AMS). In NFS, plants offer an anoxic environment, nutrients and organic acids to the diazotrophic bacteria, which in exchange provide fixed atmospheric nitrogen to the host plant. This process occurs in the nodule, a root structure derived from inner or outer cortical cells, depending on whether the organ belongs to the indeterminate or determinate type, respectively (Roy et al., 2020). In AMS, hyphae of arbuscular mycorrhizal fungi (AMF) penetrate root epidermis, colonize cortical cells and form highly branched structures called arbuscules. This ancestral interaction allows plants to improve the use of natural soil resources including macronutrients (e.g., phosphorus and nitrogen) and water, and to better respond to abiotic and biotic stresses (Gianinazzi et al., 2010; Wipf et al., 2019).

The legume-rhizobia symbiosis is initiated by a molecular dialogue. Plant roots exude flavonoids and betaines that induce the expression of bacterial *nod* genes (Peters et al., 1986; Redmond et al., 1986; Dénarié and Cullimore, 1993). *nod* genes encode nodulation factors (NFs) that trigger several molecular responses in legumes, including intracellular Ca^2+^ oscillation (Charpentier and Oldroyd, 2013), root hair (RH) deformation, and NF-induced signaling genes (Geurts et al., 2005).

A conceptual signaling cascade, the so-called ‘*common symbiosis signaling pathway’* (CSSP), is shared between the NFS and AMS (Oldroyd, 2013). The CSSP signaling is triggered by the perception of NFs (rhizobia) and Myc (mycorrhizal fungus) factors at the plasma membrane, leading to nuclear calcium spiking. In the model legume *Lotus japonicus*, this pathway includes genes such as the *SYMRK* receptor-like kinase, the nucleoporins *NUP85*, *NUP133* and *NENA*, cationic channels *CASTOR* and *POLLUX*, located on the nuclear envelope and a nuclear calcium- and calmodulin-dependent kinase, *CCaMK* and its substrate *CYCLOPS* (Genre and Russo, 2016; Roy et al., 2020). SYMRK interacts with the NF receptors in Lotus and is required for the Myc Factor induced calcium spiking. CCaMK interprets the rhythmic nuclear calcium elevations and interacts with the transcription factor CYCLOPS. In the NFS, CYCLOPS activates NIN and ERN1 that trigger expression of genes required for formation of an infection thread (IT) and nodule organogenesis. In the AMS, CYCLOPS activates RAM1, one of the best characterized mycorrhizal-induced TF, that is essential for the development of the highly branched arbuscular form of the inner cortex. RAM1 is thus involved in both nutrient uptake from, as well as carbon supply to, the fungus (Rich et al., 2017; Pimprikar and Gutjahr, 2018).

Recent evidence has raised the importance of the cytoskeleton during the formation of both symbiosis (Liu et al., 2020; Roy et al., 2020). In the NFS, reorganization of actin filaments has been related to several events, including IT formation, cell membrane remodeling, and nucleus relocation (Timmers, 2008). Additionally, it has been reported that the presence of NFs induces RH deformation as a consequence of actin filament remodeling (Cárdenas et al., 1998). Progression of the IT is directed by bacterial growth and division of the cell wall, as well as by extracellular matrix deposition and actin remodeling (Timmers, 2008). Interestingly, mutations in genes coding for proteins belonging to SCAR/WAVE complex, that regulates the actin remodeling complex, result in defects in rhizobial infection and nodule colonization (Yokota et al., 2009). In AMS, host cells undergo a drastic reorganization of cellular organelles, including the nucleus and endoplasmic reticulum (ER), as well as changes in the membrane and cell wall that allow hyphal progression through plant tissues. Actin filaments and microtubules form a dense network in arbuscule-containing cells involved in vesicle trafficking and membrane protein localization. Interestingly, the cytoskeleton is also actively reorganized in adjacent cells and in cells contacting the intercellular hyphae (Genre and Bonfante, 1998).

In the past years, the importance of Rho GTPases as key elements for the rearrangement of the cytoskeleton during symbiotic interactions was demonstrated (Yalovsky et al., 2008; Fu, 2010). GTPases hydrolyze nucleotide guanosine triphosphate (GTP) to guanosine diphosphate (GDP) and are functioning as a molecular switch in various signaling pathways (Yang, 2002). In beans, GTPases were demonstrated to participate in vesicle trafficking involved in polar growth of RHs (Blanco et al., 2009). Another group of small GTPase proteins called ROPs (rho of plants) have been described to regulate several biological processes such as endocytosis, exocytosis, auxin response, secondary cell wall, response to oxidative stress, and response to bacteria and fungi (Rivero et al., 2019).

Rho of plants belong to a highly conserved multigenic family and act as molecular switches, oscillating between an active state (bound to GTP) and inactive state (bound to GDP). Activation induces a conformational change that allows interaction with their molecular effectors. ROPs are regulated by other protein families such as the activators ROPGEF (GDP/GTP Exchange Factors), the inactivators ROPGAP (GTPase Activating Proteins) and the chaperon proteins ROPGDI (GDP Dissociation Inhibitors) (Rivero et al., 2019). ROPGEFs catalyze GDP/GTP exchange and activate ROPs, while ROPGAPs increase ROP-GTP hydrolysis. Additionally, GDIs participate in determining ROP subcellular localization and protein half-life (Feiguelman et al., 2018). From a structural point of view, ROPs contain a catalytic N-terminal domain, where GTP, GDP, and effectors interact. The C-terminal domain is a hypervariable region containing prenylation or geranylation sites. These post-translational modifications depend on the hypervariable sequence and are used to classify ROPs as ROP type I or ROP type II proteins (Feiguelman et al., 2018).

It has been proposed that ROPs are involved in the establishment of legume-rhizobia interactions (Ke et al., 2012, 2016; Liu et al., 2020). During the NFS, MtROP10 from *Medicago truncatula* and ROP6 from *L. japonicus* interact directly with NF receptors, suggesting a possible participation of ROPs in the NFS signaling pathway (Ke et al., 2012; Lei et al., 2015). Additionally, it was recently reported that ROP6, one of the 10 ROP members in *L. japonicus*, is important for the formation and maintenance of the IT through its activation by the GEF protein SPIKE1. ROP6 is also related to the NFS *via* its interaction with the clathrin heavy chain 1 (CHC1) protein, stimulating the early nodulation gene expression and rhizobial infection (Wang et al., 2015). Recently, two studies showed that GEF2, phosphorylated by NFR1/LYK3, controls the NFS in soybean and *M. truncatula* through the activation of ROP9 (Wang et al., 2021). In soybean, this regulation occurs through the association of ROP9 with a scaffold protein Receptor for Activated C Kinase (RACK1), which plays a pivotal role in *Phaseolus vulgaris* NFS (Islas-Flores et al., 2012).

However, no evidence for the role of ROPs in the AMS has been described to date, suggesting that ROPs act independently of CSSP. To investigate whether other ROP family members could be part of the NFS regulation and to better understand the role of ROPs in mutualistic symbioses, we decided to further study the potential role of *ROP1*, *ROP3*, and *ROP10* during the NFS and the role of *ROP3* during AMS in *L. japonicus*. We found that *ROP3* is involved in the establishment of the NFS and that this gene is not required for the AMS in *L. japonicus*.

## Materials and Methods

### Biological Material

*Lotus japonicus* mutant lines were obtained from the LORE1 mutant database^1^ (Małolepszy et al., 2016). *rop1, rop3*, and *rop10* ID mutant lines were 30000786, 30000537, and 30058643, respectively. Wild-type Gifu wild-type seeds and *Mesorhizobium loti* (strain MAFF303099) were kindly provided by Krzysztof Szczyglowski’s laboratory. The mycorrhizal fungus *Rhizophagus irregularis* DAOM 197198 was obtained from Agronutrition (Carbonne, France).

### Genotyping

To confirm the genotype of LORE1 insertion mutants, genomic DNA from each mutant was extracted and amplified by PCR using specific primers (Supplementary Table 1) as previously described (Urbański et al., 2012). Genomic DNA was extracted from each mutant and Gifu wild-type young trifoliate leaves according to Alonso and Stepanova (2015). DNA concentration was evaluated by UV spectrophotometry (NanoDrop^TM^, Thermo Fisher Scientific^TM^, United States).

### Construction of Binary Vectors for Plant Transformation

For functional complementation, a 2,000 bp fragment, located prior to the start codon of the *ROP3* promoter, was amplified from genomic DNA using primers described in the Supplementary Table 1. The amplicon was then purified, digested with restriction enzymes *Aat*II and *Sal*I, and inserted into the binary vector PC-GW (Dalal et al., 2015) using DNA ligase (#EL0011). The coding sequence of *ROP3* was synthesized by GenScript^2^, digested with *Aat*II and *Xba*l, and ligated into the binary vector PC-GW mCherry (Dalal et al., 2015).

### Plant Transformation and Functional Complementation

*Agrobacterium rhizogenesis* AR10 strains containing the binary vectors were used to transform *L. japonicus* roots. Seeds were germinated for a week in B&D medium (Broughton and Dilworth, 1971) supplemented with 1.2% agar and were kept in the dark to elongate hypocotyls. After 2 days, hypocotyls were punched with a needle covered with *A. rhizogenesis* AR10. Plants were transferred to a new B&D plate and kept in the dark for 24 h, and then transferred to a growth chamber for 2 weeks (28% humidity, 22°C and 16/8 h photoperiod). Transformed roots were selected using mCherry fluorescence and transferred to vermiculite.

To perform functional complementation, Gifu wild-type, and *rop3* plants were transformed with the empty vector and the binary vector containing the promoter and the coding region of *ROP3* (*pROP3:ROP3*). Plants were inoculated with *M. loti* 7 days after being transferred to vermiculite to count the nodules. Results were obtained from 3 independent experiments (*n* > 15 roots).

### Plant Growth Conditions

Seeds were surface sterilized in a solution containing 75% ethanol and 0.1% SDS for 5 min. The solution was then discarded and seeds were treated with a 20% bleach + 0.1% SDS solution for 2 min and rinsed 10 times with sterile water. Afterward, seeds were incubated at room temperature under continuous shaking overnight. Seeds were then placed on Petri dishes containing wet Whatman manuscript and incubated for 1 week in a growth chamber at 21–25°C and 16/8 h photoperiod. Seedlings were transferred to pots containing vermiculite supplemented with either B&D medium (Broughton and Dilworth, 1971) with minimal nitrogen concentration (0.5 mM) for inoculated plants or B&D medium supplemented with 1 mM nitrogen for non-inoculated plants. During the first week, pots were covered with a plastic bag and gradually opened to allow proper acclimation of the plants.

### *Mesorhizobium loti* Inoculation

*Lotus japonicus* plants were inoculated with *M. loti* 7 days after transfer to pots, by pipetting 1 mL of bacteria grown 2 days in TY medium at 28°C until reaching a OD of 0.8 at 600 nm and resuspended in tap water as previously described (Pajuelo and Stougaard, 2005).

### Characterization of Symbiosis-Related Phenotypes

Infections threads (ITs), nodulation events (number of nodules and nodule primordia), microcolonies and percentage of deformed RHs were determined 7 or 21 days after inoculation (DAI). Visualization of the symbiotic bacteria was achieved through LacZ staining of the *M. loti* MAFF303099 carrying the *hemA:lacZ* reporter gene (Wopereis et al., 2000). *LacZ* staining was performed as previously described (Díaz et al., 2005). Roots were mounted in water and observed under a 20x objective on a Zeiss Axioskop 2 (Zeiss^TM^, Germany) coupled to a Zeiss AxioCam MRc camera. IT and nodulation events were determined by counting total IT or nodulation events divided by the length of the primary root. All values correspond to *n* > 15 roots per line and are derived from three independent experiments.

### Gene Expression Analysis

Total RNA was isolated from roots at 7 DAI for the *M. loti* interaction and 2 month post inoculation (MPI) for the *R. irregularis* interaction. RNA was isolated using the Plant/Fungi Total RNA Purification Kit according to the manufacturer’s instructions (NORGEN BIOTEK CORP., Canada) and treated with RNase free DNase I, (Thermo Fisher Scientific^TM^, United States). The concentration of purified total RNAs were estimated using a UV spectrophotometer (NanoDrop^TM^, Thermo Fisher Scientific^TM^, United States). First-strand cDNA was synthesized using 1 μg of total RNAs and SCRIPT cDNA Synthesis Kit according to the manufacturer’s instructions (Jena Bioscience, Germany). Real-time quantitative PCR was conducted with Maxima SYBR Green/ROX qPCR Master Mix (2X) (Thermo Fisher Scientific^TM^, United States) and 10 μM gene-specific primers (Supplementary Table 1). Each PCR (10 μL final volume) contained 4 μL of cDNA, 1 μl of primers mixture, and 5 μL of Maxima SYBR Green/ROX qPCR Master Mix. Thermocycling conditions were as follows: an initial holding stage of 2 min at 50°C, followed by 15 min at 95°C, then 40 cycles of denaturation for 15 s at 95°C, annealing for 30 s at 62°C and extension for 30 s at 72°C, with a final melt gradient starting from 60°C and heating to 95°C at a rate of 0.3°C s^–1^. The real-time PCRs were carried out in the StepOnePlus^TM^ Real-Time PCR System (Applied Biosystems^TM^, United States). The PCR efficiency was confirmed by establishment of a standard curve. The *PROTEIN PHOSPHATASE 2A* (*PP2A*), UBIQUITIN (*UBC*), and *ATP SYNTHASE* (*ATPs*) genes served as internal controls. Three independent experiments each with 3 biological replicates were performed for each tested condition.

### *Rhizophagus irregularis* Inoculation

*Lotus japonicus* seeds were surface-sterilized (2.5% KClO, 10 min), then rinsed with sterile deionized water several times and soaked in sterile deionized water overnight. Seeds were pre-germinated on autoclaved sand (121°C, 30 min) at 25°C for 24 h and then grown in the dark at room temperature for 72 h. One plantlet was transplanted per 1.5-L pot filled with an autoclaved (121°C, 30 min) quartz sand (diameter < 4 mm, Kaltenhouse, France): zeolithe (diameter 1– 2.5 mm, Symbiom, Czechia) mixture (1:1, *v:v*). To establish mycorrhizal and rhizobial symbioses, plantlets were individually inoculated with the arbuscular mycorrhizal fungus (AMF) *R. irregularis* DAOM 197198 (1 ml of a suspension of 100 spores/mL). For the controls (non-inoculated plantlets), the same amount of autoclaved inoculum was added to the mixture. Plants were grown under controlled conditions (12 h of light at 24°C and 12 h of dark at 22°C) and weekly fertilized with a modified Hoagland solution (P concentration: 0.5 mM; N concentration: 6 mM) (Calabrese et al., 2019). After 2 months of growth post-inoculation (vegetative state), plants were gently dug out. Roots were divided into two subsamples; one of 100 mg was snap-frozen in liquid nitrogen and stored at −80°C for gene expression analysis and one was used to determine the degree of AMF root colonization. Remaining root samples and shoot materials were dried at 60°C for 48 h and weighed. Root subsamples were immersed in 10% KOH and stored in 20-ml glass tubes at 4°C overnight. Roots were rinsed under tap water and immersed in 2% HCl at room temperature for 1 h. Roots were rinsed under tap water, immersed in 0.05% trypan blue and stored at 4°C overnight. Roots were rinsed again under tap water and destained in lactic acid glycerol (3:1). Total root colonization was measured using the method described by Trouvelot et al. (1986).

### Phosphorus, Nitrogen, and Carbon Quantification

To measure N uptake by plants, 15N-enriched ammonium nitrate was applied once in each pot at 1 month post-inoculation (15NH_4_15NO_3_, > 98% 15N; Sigma Aldrich, St. Louis, United States) to reach δ 15N = + 4450, corresponding to an enrichment of 2% (calculated value using isotopic abundance of the unlabeled and 15N-enriched ammonium nitrate and their molar ratio in the liquid fertilizer). Dry shoot and root materials were ground separately in tungsten tubes using tungsten carbide balls in a Retch MM301 vortexer (Retch GmbH & Co., Haan, Germany). Total N, 15N and C aliquots of 2 mg were weighed for elemental analyses, and their concentrations were determined using an ANCA elemental analyzer/mass spectrometer (Wellience, Dijon, France). To measure phosphorus (P) content of plants, dry leaf material (20 mg) was ashed at 550°C for 8 h. The residue was dissolved in 2 ml H_2_O and 100 μl HCl (32%). One ml of the solution was then transferred to a 2-ml Eppendorf tube and centrifuged at 12,000 rpm for 10 min in a benchtop centrifuge. One hundred μl of the supernatant were transferred into a well of a 96-wells microtiter plate. Phosphorus concentration was then measured by the malachite green method (Ohno and Zibilske, 1991).

### Statistical Analyses

All graphs and statistical analysis were performed on GraphPad Prism 9. An analysis of variance (ANOVA) was performed on the normalized gene expressions, followed by Tukey’s HSD test for separating the means. Differences between means of variables were analyzed by Kruskal-wallis test, followed by *post hoc* Dunn’s Multiple Comparison Test. A probability of *P* ≤ 0.05 was considered as significant.

## Results

### The *rop3* Mutant Shows Defects in the *Lotus japonicus*- *Mesorhizobium loti* Symbiosis but Not in Root Growth

**FIGURE 1:**
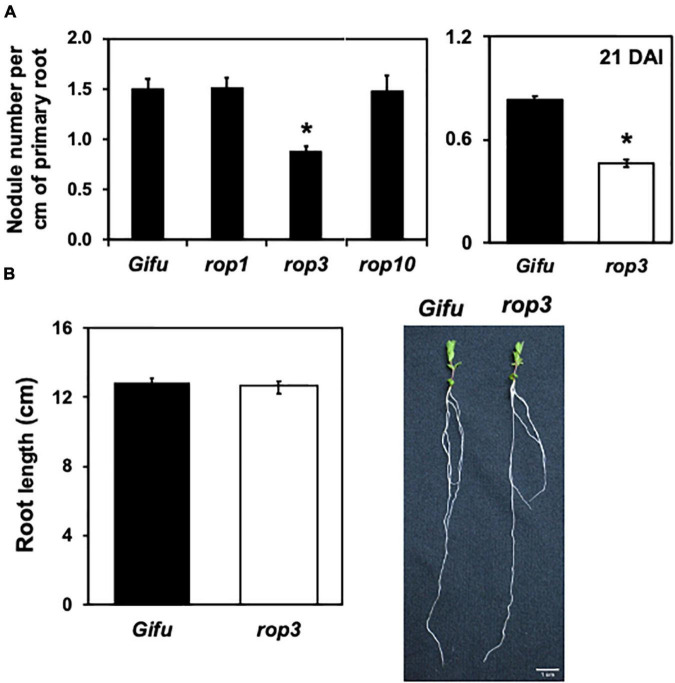
*rop3* mutant presents defects in the *L. japonicus-M. loti* symbiosis. *Lotus japonicus* Gifu wild-type and the indicated mutant lines were inoculated with *M. loti* and **(A)** number of nodules were quantified at 7 DAI (left) and at 21 DAI (right). **(B)** Primary root length of *L. japonicus* plants grown under optimal conditions were measured, and pictures of representative Gifu wild-type and *rop3* plants are shown. Bars represent mean values (± SD) of 3 independent experiments (*n* > 15). The asterisk indicates statistical significance between the indicated mutant and Gifu wild-type according to one way ANOVA test and a *post hoc* analysis (*p* < 0.05).

The genome of *L. japonicus* contains 10 *ROP* genes and, to date, few insertional mutants have been generated for these genes, including mutants in *ROP1*, *ROP3*, *ROP6*, and *ROP10*. To assess a potential role of ROPs other than *ROP6* (Ke et al., 2012, 2016) during the NFS between *L. japonicus* and *M. loti*, we quantified the infection events in *rop1*, *rop3*, and *rop10* mutants. Seven DAI, *rop3* showed a reduction of about 40% in nodules number compared to the wild-type plant (Gifu wild-type), while *rop1* and *rop10* were not different from wild type plants (Figure 1A and Supplementary Figure 1). These results indicate that from the *ROP* gene mutants analyzed, only *rop3* presented defects in the NFS. To assess the effect of a delay or a real defect in nodule formation, we counted the number of nodules per cm of primary root at 21 DAI. We observed significantly reduced nodulation in *rop3* compared to Gifu wild-type plants, suggesting a strong effect on nodule formation (Figure 1A). The insertion in the *rop3* mutant line is located in the 5′UTR region (Supplementary Figure 2A). In order to confirm that the transposon insertion in *ROP3′s* 5′UTR altered its expression, we first compared *ROP3* transcript accumulation by qRT-PCR in Gifu wild-type and the *rop3* mutant line. Almost no accumulation of transcript was observed in the insertional mutant as compared to Gifu wild-type, confirming that the insertion impairs *ROP3* expression (Supplementary Figure 2B). Additionally, as those insertions lines contained more than one insertion and to ensure that the observed phenotype was due only to the loss of *ROP3* expression, we performed a functional complementation assay. A native copy of *ROP3* was reinserted into the *rop3* background and the complemented *rop3* plants regained the ability to produce a number of nodules similar to Gifu wild-type plants transformed with an empty vector (Supplementary Figure 2C). This confirmed that the observed phenotype is associated with the lack of *ROP3* expression.

Rho of plants are known to regulate multiple biological processes (Rivero et al., 2019). Before further characterization of the role of *rop3* during NFS, we wanted to ensure that it would not cause any defect in plant root development. *L. japonicus* Gifu wild-type and *rop3* mutant lines were grown under optimal conditions (soil supplemented with nitrogen) and their primary root length was measured. No significant differences were observed between Gifu wild-type and *rop3* plants (Figure 1B). These results suggest that *ROP3* may not be involved in root development but rather during the symbiotic interaction.

### Bacterial Infection and Progression Within the Plant Is Hindered in *rop3*

**FIGURE 2:**
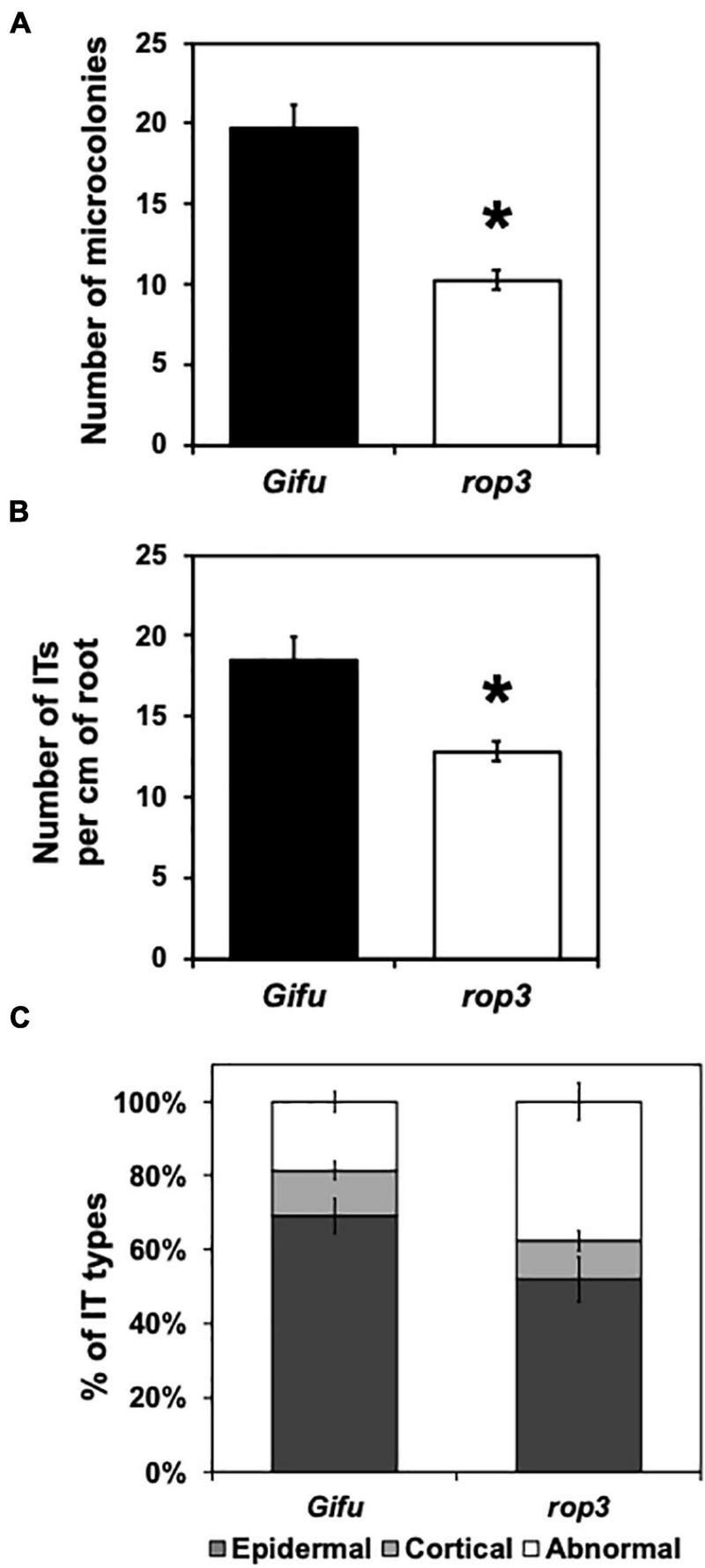
Infection thread progression are modified in the *rop3* mutant. Number of microcolonies **(A)**, infection thread per cm of root **(B),** and Infection thread types **(C)** were quantified on Gifu wild-type and *rop3* plants at 7 DAI with *M. loti*. Bars represent mean values (± SD) of 3 independent experiments (*n* > 15). The asterisk indicates statistical significance between *rop3* and Gifu wild-type according to one way ANOVA test and a *post hoc* analysis (*p* < 0.05).

Rhizobial microcolonies in the infection chamber of RH curls, ITs as well as their progressions, were quantified and compared between Gifu wild-type and *rop3* plants at 7 DAI with *M. loti*. We measured a reduction of about 50% of the number of microcolonies in *rop3* plants compared to Gifu wild-type (Figure 2A and Supplementary Figure 3). Additionally, *rop3* showed a significant reduction in IT number per cm of root (Figure 2B). Moreover, *rop3* produced less cortical IT than Gifu wild-type and exhibited abnormal IT growth arrest in the epidermis and thus not reaching cortical cells (Figure 2C and Supplementary Figure 3). These results indicate that ROP3 play an important role in the entry and progression of the bacteria into the plant roots.

### Root Hair Deformation in Response to Symbiotic Bacteria Is Misregulated in *rop3* Mutant

**FIGURE 3:**
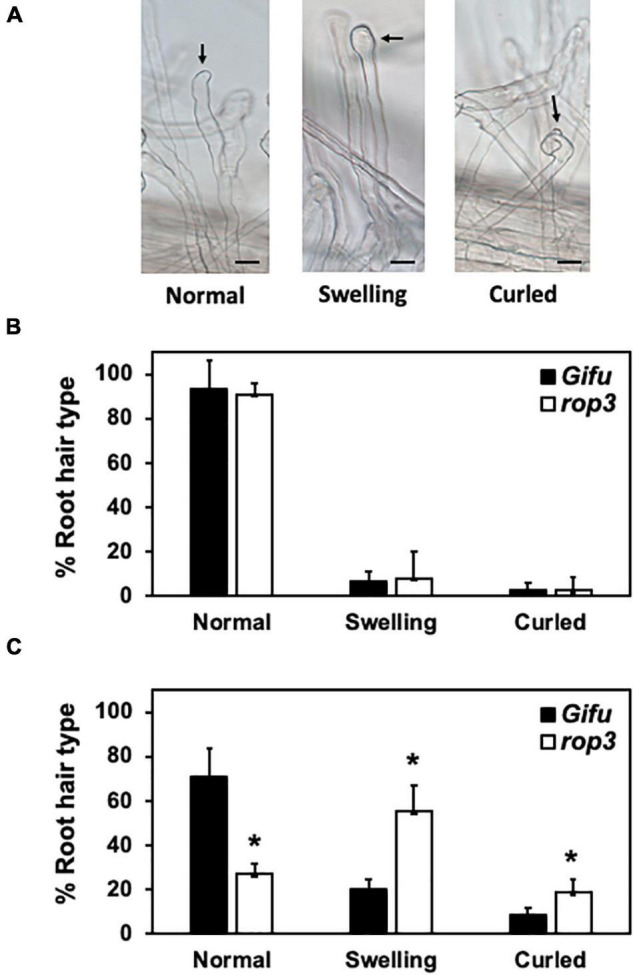
*rop3* plants showed a misregulation of RH deformation after interaction with *M. loti*. RHs deformation types were counted in *rop3* and Gifu wild-type plants. Representative pictures of RH types evaluated in Gifu wild-type and *rop3* plants, arrows point the site of interest, black bar = 40 μm **(A).** RH deformation types without bacteria inoculation **(B)** and at 7 DAI with *M. loti* **(C)**. Bars represent mean values (± SD) of 3 independent experiments (*n* > 15). The asterisk indicates statistical significance between *rop3* and Gifu wild-type according to one way ANOVA test and a *post hoc* analysis (*p* < 0.05).

To further characterize the phenotypic alterations observed in *rop3*, we compared RHs deformation in *rop3* and Gifu wild-type plants (Figure 3A), with and without rhizobial inoculation. In non-inoculated plants, *rop3* and Gifu wild-type shared the same low percentage of deformed RHs (Figure 3B). However, at 7 DAI, a fraction of Gifu wild-type RHs was deformed in response to the bacteria, but the majority was not deformed and conserved their normal shape. In contrast, *rop3* exhibited a greater number of deformed RHs, both swollen and curled (Figure 3C). The absence of ROP3 did not seem to impede RH deformation in response to symbiotic bacteria but rather increased its frequency and extent. These results indicate that ROP3 does not take part in normal RH formation or growth but might be involved in regulating RH deformation responses.

### The Benefits of Arbuscular Mycorrhizal Symbiosis Are Not Altered in *rop3* Mutant

**FIGURE 4:**
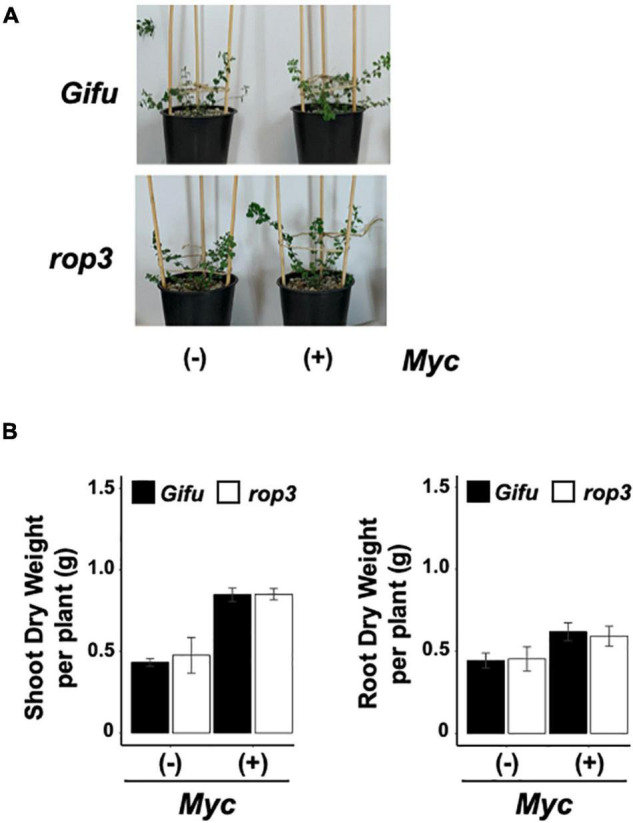
ROP3 does not play a role in AM symbiosis in *L. japonicus*. **(A)** Development of Gifu wild-type and *rop3* plants inoculated with *R. irregularis*. Pictures of representative plants are shown. **(B)** Shoot and root dry weight were determined for Gifu wild-type and *rop3* plants with and without inoculation. Bars represent mean values (± SD) of 3 independent experiments (*n* = > 15).

**FIGURE 5:**
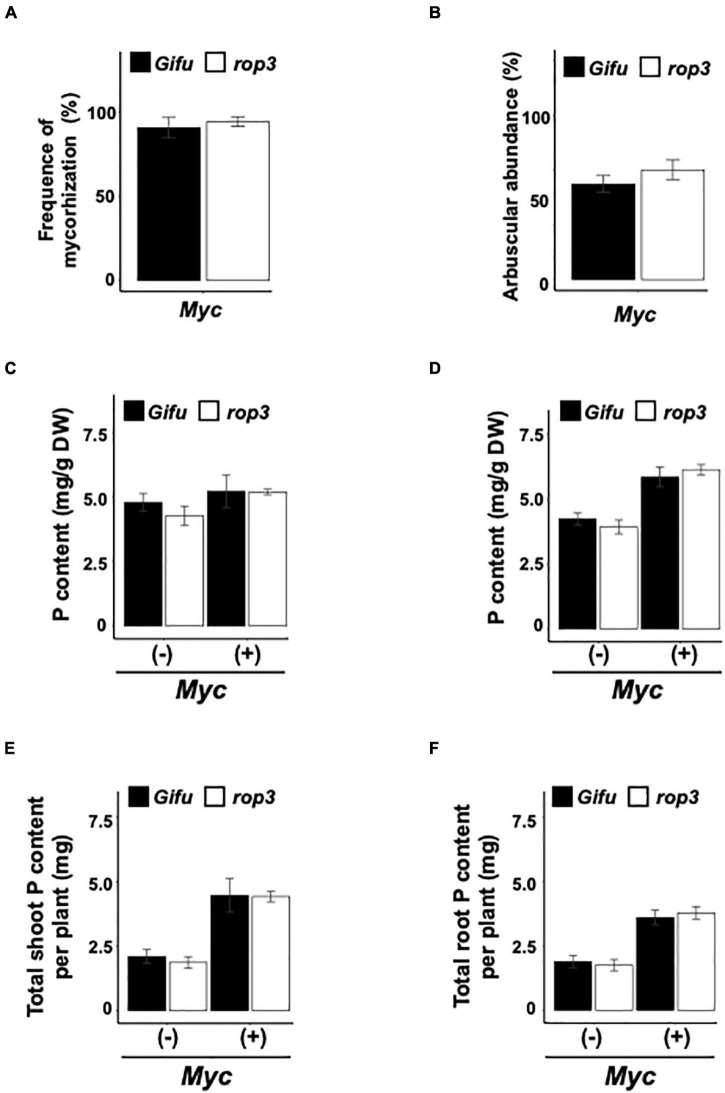
Arbuscular mycorrhizal symbiosis is not altered in the *rop3* mutant. **(A)** Frequency of mycorrhization, **(B)** arbuscular abundance, **(C)** phosphate contents in dried shoots, **(D)** roots, **(E)** total phosphate contents of shoots, and **(F)** roots were determined in Gifu wild-type and *rop3* plants inoculated with *R. irregularis*. Bars represent mean values (± SD) of 3 independent experiments (*n* = > 15).

Based on the role of *ROP3* in nodulation, we hypothesized that the mutation should also impact the AMS. Shoot and root dry weight were significantly increased when both Gifu wild-type and *rop3* plants were inoculated with *R. irregularis* (Figures 4A,B). Frequencies of mycorrhization (F%) and arbuscule abundance (A%) in the root system were similar in Gifu wild-type and *rop3* plants (Figures 5A,B). To assess the efficiency of the mycorrhizal symbiosis, P elemental analysis was performed. P contents and P concentrations in shoots and roots were significantly higher in mycorrhizal plants compared to non-mycorrhizal plants in both genotypes (Figures 5C–F). We analyzed the expression of three plant mycorrhizal marker genes *AMT2.2, PT4*, and *PT8* genes (Guether et al., 2009; Casieri et al., 2013). Marker genes were induced by the infection with *R. irregularis*, without significant difference between Gifu wild-type and *rop3* (Supplementary Figure 4). In both Gifu wild-type and *rop3* plants, inoculation with *R. irregularis* increases N and P content in plant tissues and increased C assimilation compared to control plants (Supplementary Figure 5). However, no significant differences were observed between Gifu wild-type and *rop3* plants.

### Expression of Nodulation Signaling Pathway Genes Is Altered in *rop3* Mutant

**FIGURE 6:**
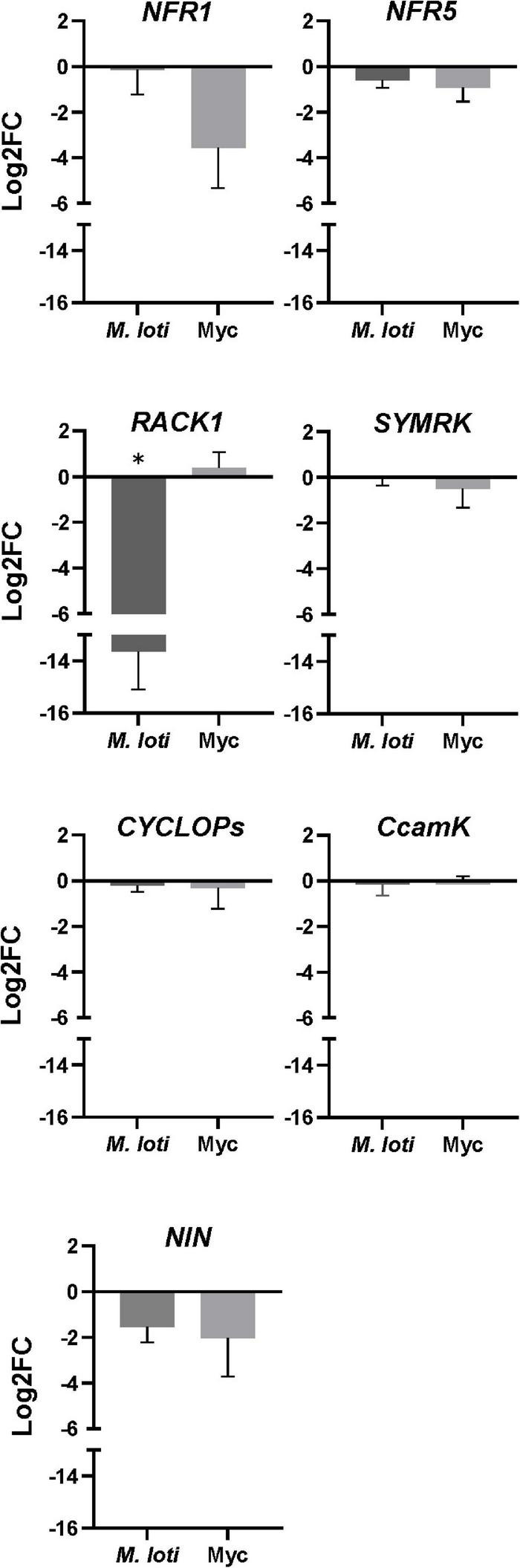
RSP but not CSSP gene expression is altered in the *rop3* mutant. Expression analysis of indicated symbiotic genes was performed by qRT-PCR for Gifu wild-type and *rop3* mutant plants inoculated either with *M. loti* (at 7 DPI) or with *R. irregularis* (at 2 MPI). Data show the ratio of Log2FC values obtained from *rop3* vs. Gifu wild type plants. Three independent experiments each with 3 biological replicates were performed. The asterisk indicates statistical significance between *rop3* and Gifu wild-type according to one way ANOVA and a *post hoc* analysis (*p* < 0.05).

In legumes, nodule formation is regulated by a complex signaling pathway, including genes from the CSSP and genes specific to the rhizobial signaling pathway (RSP) (Roy et al., 2020). We hypothesized that the expression of some of these genes might be altered in *rop3*, revealing possible explanations for the different phenotypes observed between NFS and AMS. Therefore, we analyzed the relative expression of representative genes of RSP and CSSP in Gifu wild-type and *rop3* plants during both symbioses (Figure 6). As expected, *SYMRK*, *CYCLOPS*, and *CCAMK* expression was not impacted by both plants and interactions, which suggests that the reduced number of nodules in *rop3* is not due to an alteration of CSSP gene expression. In contrast, expression of RSP-related genes (*NFR1, NFR5, RACK1*, and *NIN*) was altered in *rop3* compared to Gifu wild-type plants. Particularly, the expression of *RACK1* is highly reduced in *rop3* during the NFS and not in the AMS. The expression of two other RSP genes, *NFR5* and *NIN*, was also altered in *rop3* compared to Gifu wild-type, but in a similar way in both interactions. Hence, the symbiotic phenotype of *rop3* could be due to a functional CSSP but an altered RSP.

## Discussion

Biological nitrogen fixation in legumes is regulated by a set of well-described genes, from NF perception to nodule senescence (Roy et al., 2020). One of the first step of rhizobial root infection is the deformation of RHs that allows symbiont penetration in the roots. Recently, Liu et al. (2020) revealed that the absence of ROP6 leads to a reduction of infection events and IT progression in the root. In soybean and *M. truncatula*, ROP9 has also been reported to be a key player of the RSP (Gao et al., 2021; Wang et al., 2021). Here, we show that ROP3, in contrast to ROP1 and ROP10, whose corresponding mutants did not display an altered symbiotic phenotype, takes part in the *L. japonicus*-*M. loti* symbiosis. ROP3 may play a role in the bacterial infection and then progression within the root. However, in contrast to ROP6, ROP9 and homologs, ROP3 does not seem to play a role in RH formation or growth, but seems to participate in RH deformation in response to rhizobia. The lower number of ITs able to reach the root cortex in *rop3* mutant plants also indicates that ROP3 might be involved in the polarized growth of ITs following rhizobial infection. Additionally, *rop3* mutant shows a reduction in the number of detectable microcolonies trapped in the RH curl that could be due to a modification of the infection chamber conditions, a consequence of altered RH deformation, or inhibition of bacterial growth and/or division. The increased RH deformation may be link to a compensatory mechanism, as previously observed in other symbiosis-deficient mutants such as *ccamk* (Maekawa-Yoshikawa and Murooka, 2009).

In this context, we evaluated whether ROP3 might be involved in the signaling pathway in response to the rhizobial NFs. We showed that alteration of *ROP3* accumulation does not affect transcript accumulation of representative CSSP genes (Roy et al., 2020), suggesting that ROP3 is not involved in the CSSP. This result is congruent with the lack of an altered AMS phenotype in *rop3* mutant. Furthermore, we analyzed the expression profile of RSP genes in the two genotypes and symbioses. The gene most differentially expressed in *rop3* during the NFS and AMS was *RACK1.* During NFS, RACK1 has been identified as an interactor of GmROP9 in soybean, depending on the activation by the NFR1/NFR5/GEF2 complex (Gao et al., 2021), and it has been shown to be an important element of the signal transduction pathway downstream of NF detection in common bean (Islas-Flores et al., 2012). Up to date, there is no report on the implication of RACK1 in the AMS. In our study, the drastic low accumulation of *RACK1* transcripts in *rop3* is specific to the NFS, and was not found during the AMS. This phenomenon could be due to an indirect regulation of *RACK1* transcription in consequence of a misactivation by the NFR1/NFR5/GEF2 complex, and a regulation loop after NF perception, but not after Myc factor perception by the corresponding receptor complex. Like GmROP9, ROP3 could be an interactor of RACK1, and this could explain the partial nodulation in *rop3* mutant plants. We suggest that ROP3 can be compensated by other ROPs such as ROP9. In parallel, the decrease of *NFR5* and *NIN* expression may explain the reduced number of nodule formation, probably by limiting the signal transduction to SYMRK and the proper transcriptomic response, downstream NF signaling.

Rho of plants GTPases are known to be involved in vesicle trafficking, actin organization and maintenance of reactive oxygen species (ROS) as well as Ca^2+^ signaling (Bloch et al., 2011), which are essential for the NFS or the AMS (Roy et al., 2020). ROP6 interacts with the NF receptor NFR5 and is involved in the establishment of the rhizobial symbiosis (Ke et al., 2012). We could assume that ROP3, like ROP6, might also directly associate with NFR5 to initiate the NFS signaling pathway. The control of polar growth of both the RH as well as the IT by ROP3 might also be achieved through its interaction with SPIKE1, a GEF protein known to activate ROPs, similarly to what was observed with ROP6 (Liu et al., 2020). ROP6 and ROP3 might share the same activator, SPIKE1, but might trigger a different downstream response by interacting with different effectors. Alternatively, ROP3 could be associated with other receptor-like kinases involved in the perception of rhizobia, like the exopolysaccharide receptor, EPR3 (Kawaharada et al., 2015). This hypothesis would explain why *rop3* plants can still form nodules and grow like the wild type. ROPs regulate cell growth and shape by reorganizing the actin filaments that direct cytoplasmic stream and vesicle trafficking. We suggest that ROP3 might participate in this process and that its absence impairs proper RH deformation in response to rhizobia and/or the invagination of the plant plasma membrane. It will be important to assess if ROP3 plays a role in the reorganization of actin filaments in response to rhizobia or NFs, known to be regulated by the SCAR/WAVE-ARP2-3 complex (Yokota et al., 2009; Hossain et al., 2012; Qiu et al., 2015).

Additionally, spatiotemporal regulation of ROS was shown to be indispensable to the proper establishment of the NFS, as the decrease of ROS level prevents RH curling as well as formation and progression of ITs (Cárdenas et al., 2008; Damiani et al., 2016; Engelhardt et al., 2020). ROS are transiently produced during rhizobial infection in legumes. Various NADPH oxidases, also named respiratory burst oxidase homologs (Rbohs), have been associated with ROS generation (Damiani et al., 2016). Some ROPs like OsRAC1 have been demonstrated to directly interact with RbohB proteins like OsRbohB (Engelhardt et al., 2020). This interaction, which supports NADPH oxidase enzyme activity, demonstrates the regulatory role that ROPs play in the production of ROS (Engelhardt et al., 2020).

Even though ROPs have been associated with several characterized signaling proteins involved in symbioses, our knowledge is still fragmentary, and we are far from assembling all the pieces of the puzzle. Various genes coding for ROPs have been conserved during evolution (10 in *L. japonicus*) and this raises the question of their specialization or functional redundancy. It is likely that some might have evolved to perform specific tasks, and this would explain why some ROP genes could play such a pleiotropic role in plant development. Each ROP might have a different affinity for their multiple regulators like ROPGEFs, GAPs or GDIs as well as with their upstream and downstream interactors (Engelhardt et al., 2020).

Our results confirm that ROP3 is involved in the establishment of the NFS but not of the AMS, and therefore does not play a role in the CSSP. P analysis revealed that *rop3* mutation does not functionally impact the AMS (Figure 6). In the AMS, ROP3 or others ROPs might have a specialized function not yet highlighted. Kiirika et al. (2012) showed that silencing *ROP9* in *M. truncatula* results in a stimulation of the infection by AM fungus and a fungal pathogen but has a negative effect on the rhizobial symbiosis (Kiirika et al., 2012). The defect of ROS signaling and defense mechanisms enhanced fungal colonization in roots but not arbuscular frequency (Nath et al., 2016). In our study, we investigated a plant-AMF interaction under undisturbed conditions during inoculation that may lead to the establishment of compensation mechanisms by both the plant and fungus. A more complex system involving stress conditions or double inoculation with *Sinorhizobium* or even a rhizobial community would be suitable to explore the role of ROP3 in plant-microorganism interactions. However, *rop3* mutant plants exhibited a strong phenotype during the NFS suggesting that ROP3 is related to the specific RH rearrangements during rhizobial infection. It is tempting to speculate that the AMS is not affected in *rop3* mutant plants because ROP3 perhaps preferentially acts in RHs and AMF do not colonize roots via this cell type.

## Conclusion

In this study, we showed that ROP3 specifically plays a role in the establishment of the NFS, but not during the AMS. ROP3 seems to impact the RH rearrangement during NFS, controlling the formation of a functional infection chamber, and then allowing an efficient microcolony capture leading to the formation of a complete IT. ROP3 seems to be involved in RH rearrangement by impacting the proper accumulation of RACK1, an essential component of the NF signaling pathway.

## Data Availability Statement

The original contributions presented in the study are included in the article/Supplementary Material, further inquiries can be directed to the corresponding authors.

## Author Contributions

IG-S, RB, YC-F, JC-C, LH-C, VB-Z, AL-S, and MT performed the experiments. GH, P-EC, DW, DF, MS, and AT conceived and designed the experiments. IG-S, RB, P-EC, DW, DF, MS, and AT wrote and revised the manuscript. All authors contributed to the article and approved the submitted version.

## Conflict of Interest

The authors declare that the research was conducted in the absence of any commercial or financial relationships that could be construed as a potential conflict of interest.

## Publisher’s Note

All claims expressed in this article are solely those of the authors and do not necessarily represent those of their affiliated organizations, or those of the publisher, the editors and the reviewers. Any product that may be evaluated in this article, or claim that may be made by its manufacturer, is not guaranteed or endorsed by the publisher.

## Footnotes

1
https://lotus.au.dk/

2
https://www.genscript.com/

## Supplementary Material

Supplementary Table 1 List of used primers.

## Abbreviations

NFS: nitrogen-fixing symbiosis
AMS: arbuscular mycorrhizal symbiosis
AMF: arbuscular mycorrhizal fungi
DAI: days after inoculation
ROPs: rho of plants
IT: infection thread
RH: root hair
NFs: nodulation factors
CSSP: common symbiosis signaling pathway
RSP: rhizobial signaling pathway
GEF: guanine nucleotide exchange factor
GAP: GTPase-activating proteins
GDI: guanine nucleotide dissociation inhibitor.
