# Supplementary material for: The Lotus japonicus ROP3 Is Involved in the Establishment of the Nitrogen-Fixing Symbiosis but Not of the Arbuscular Mycorrhizal Symbiosis
Source: Front Plant Sci. 2021 Nov 12;12:696450. doi: 10.3389/fpls.2021.696450 (PMC8636059; doi:10.3389/fpls.2021.696450)
Supplement: Supplementary Table 1 — List of used primers. [file Table_1.DOCX]

**Suplementary Table 1.**

| Gene name | Primer name | Primer sequence |
| --- | --- | --- |
| *Rop1* | ROP1 LORE1 F | TGACAGTGCTGCCATCCACAACCA |
|  | ROP1 LORE1 R | TTTGCCACATTCAAGGACCACGGG |
| *Rop3* | ROP3 LORE1 F | GAGACCGTGGGGAAGGTGTTGCTG |
|  | ROP3 LORE1 R | CGGCAAAATCTCGCGGGCTAAAAG |
| *Rop10* | ROP10 LORE1 F | TGCCCCAAGATTGTACGCCCATGT |
|  | ROP10 LORE1 R | GGCCTTTGAGCTACAGAGGGGCAGA |
| *Rop3* | LjRop3 RT F | TGTCCTTCAACCACCTAAGCAA |
|  | LjRop3 RT R | GAAGGGGAGGGAAATACAGG |
|  | LjRop3 prom F | ACTGGTCGACAGTGTGGGTGCTATCCTCCT |
|  | LjRop3 prom R | ACTGGACGTCCTTTTCTTCAACAAAACAAAACAAAACG |
|  | LORE 1 P2 | CATGGCGGTTCCGTGAATCTTAGG |
| *NIN* | LjNIN RT F | TGGATCAGCTAGCATGGAAT |
|  | LjNIN RT R | TCTGCTTCTGCTGTTGTCAC |
| *NFR1* | LjNFR1 qPCR F | CACAGAACCGCAGGTCTAGC |
|  | LjNFR1 qPCR R | CTGCACTACTAGAGGCATTACCA |
| *NFR5* | LjNFR5 RT F | CTCACCGGAAGGAAAGCCAT |
|  | LjNFR5 RT R | ATTTCAGCCATGGAGGGTCG |
| *SYMRK* | LjSYMRK RT F | AACCTACTTTCAGCAATACAGCA |
|  | LjSYMRK RT R | TCTCTTTGCAGGTTCCCCAT |
| *CYCLOPs* | LjCYCLOPS RT F | CTCTCTGCGAAAGCTCATGGA |
|  | LjCYCLOPS RT R | CTCCCTTGTCATCTGAAACAGC |
| *CCAMK* | LjCCAMK RT F | TGTGGAGGTGCTGAAAGCAA |
|  | LjCCAMK RT R | CCTTGGTGATGCACCCTGAT |
| *RACK1* | LjRACK1 RT F | GCCCTCTGCTTTAGTCCCAA |
|  | LjRACK1 RT R | TGGTGCAGTAGATAACCTTCTTCT |
| *UBC* | UBC F | ATGTGCATTTTAAGACAGGG |
|  | UBC R | GAACGTAGAAGATTGCCTGAA |
| *ATPs* | ATP F | CAATGTCGCCAAGGCCCATGGTG |
|  | ATP R | AACACCACTCTCGATCATTTCTCTG |
| *PP2a* | PP2A F | GTAAATGCGTCTAAAGATAGGGTCC |
|  | PP2A R | ACTAGACTGTAGTGCTTGAGAGGC |
| *AMT2.2* | LjAMT2.2 RT F | GGCTGCTCATGGGGAAGA |
|  | LjAMT2.2 RT R | CCACCACCACGCTTGTTC |
| *PT4* | LjPT4RT F | GCAGATTAAGCACGCCATGA |
|  | LjPT4RT R | GCTCACTCTCTCTCCCATCC |
| *PT8* | LjPT8RT F | TATAGTGCCGGCAGAGCTTT |
|  | LjPT8RT R | TTGTCGTCGGCTTTCTCTGT |
