# Supplementary material for: The Lotus japonicus ROP3 Is Involved in the Establishment of the Nitrogen-Fixing Symbiosis but Not of the Arbuscular Mycorrhizal Symbiosis
Source: Front Plant Sci. 2021 Nov 12;12:696450. doi: 10.3389/fpls.2021.696450 (PMC8636059; doi:10.3389/fpls.2021.696450)
Supplement: Supplementary file 2 [file Data_Sheet_1.PDF]

The *Lotus japonicus* ROP3 is involved in the establishment of the nitrogen-fixing symbiosis but not of the arbuscular mycorrhizal symbiosis.

Ivette García-Soto[1*], Raphael Boussageon[2], Yareni Marlene Cruz-Farfán[1], Jesus Daniel Castro-Chilpa[1], Liz Xochiquetzal Hernández-Cerezo[1], Victor Bustos-Zagal[1], Alfonso Leija-Salas[1], Georgina Hernández[1], Martha Torres[1], Damien Formey[1], Pierre-Emmanuel Courty[2], Daniel Wipf[2], Mario Serrano[1*] and Alexandre Tromas[1,3*]

Page 1: Supplementary Figure 1. Nodule formation in rop1, rop3 and rop10. Nodulated zone-cropped representative pictures of roots at 7 DAI of each indicated line. White bar = 1 cm.

Page 2: Supplementary Figure 2. Modification of *L. japonicus-M. loti* symbiosis is associated with a lack of *ROP3* expression. (A) Schematic representation of LORE1 insertions in *rop1*, *rop3* and *rop10* lines. (B) *ROP3* gene expression in Gifu wild-type and *rop3*. (C) The number of nodules per cm of primary root were quantified at 7 DAI on each indicated line. Bars represent mean values (± SD) of 3 independent experiments (n>15). The asterisk indicates statistical significance between the indicated line and Gifu wild-type according to one way ANOVA and a post hoc analysis ($p<0.05$).

Page3: Supplementary Figure 3. Microcolony localization and infection thread alterations. Representative pictures of trypan blue-stained RHs. Black arrows point the site of interest. From left to right: microcolony localization, epidermal IT, cortical IT and abnormal IT. Black bar = 40 µm.

Page 4: Supplementary Figure 4. Normalized expression of mycorrhizal marker genes in *L. japonicus*. Bars represent mean values (± SD) of relative gene expression of *AMT2.2, PT4* and *PT8*. Three independent experiments each with 3 biological replicates were performed.

Page 5: Supplementary Figure 5. N and C assimilation mediated by *R. irregularis*. Total nitrogen (A), total nitrogen content per g dry weight and (B) total carbon of Gifu wild-type and *rop3* inoculated plants were determined. Bars represent mean values (± SD) of 3 independent experiments (n>15).

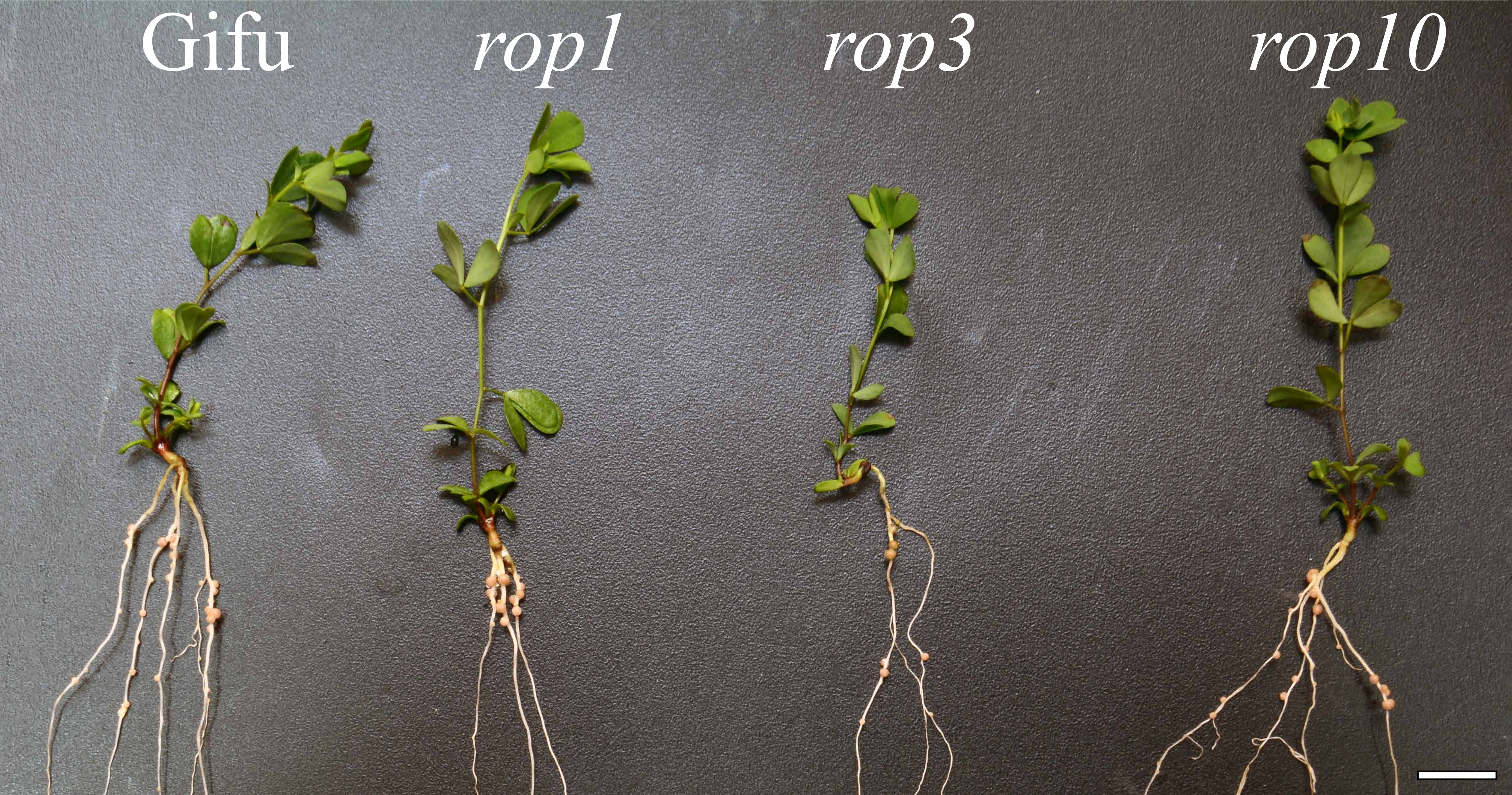
Gifu
rop1
rop3
rop10

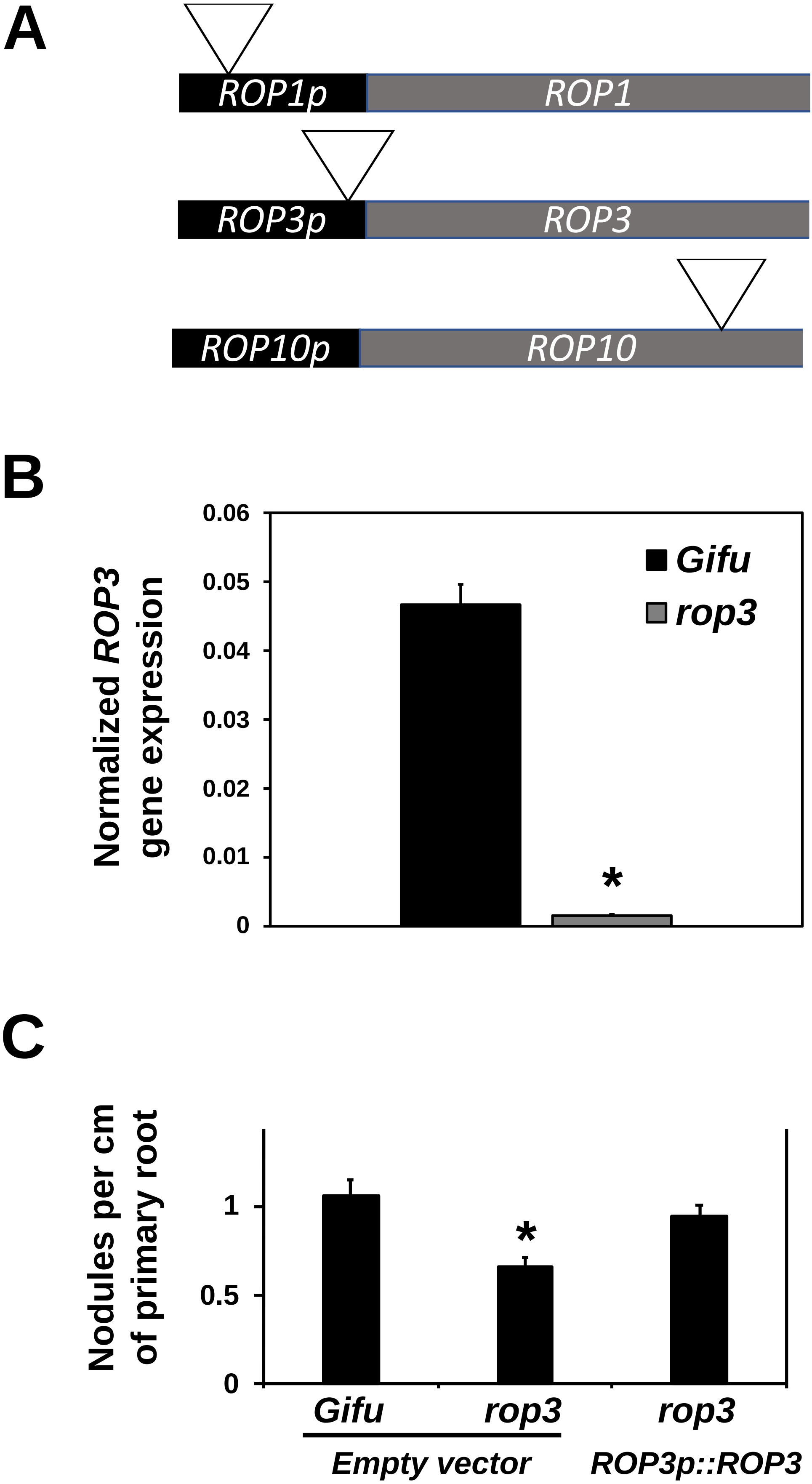

Supplementary Figure 2

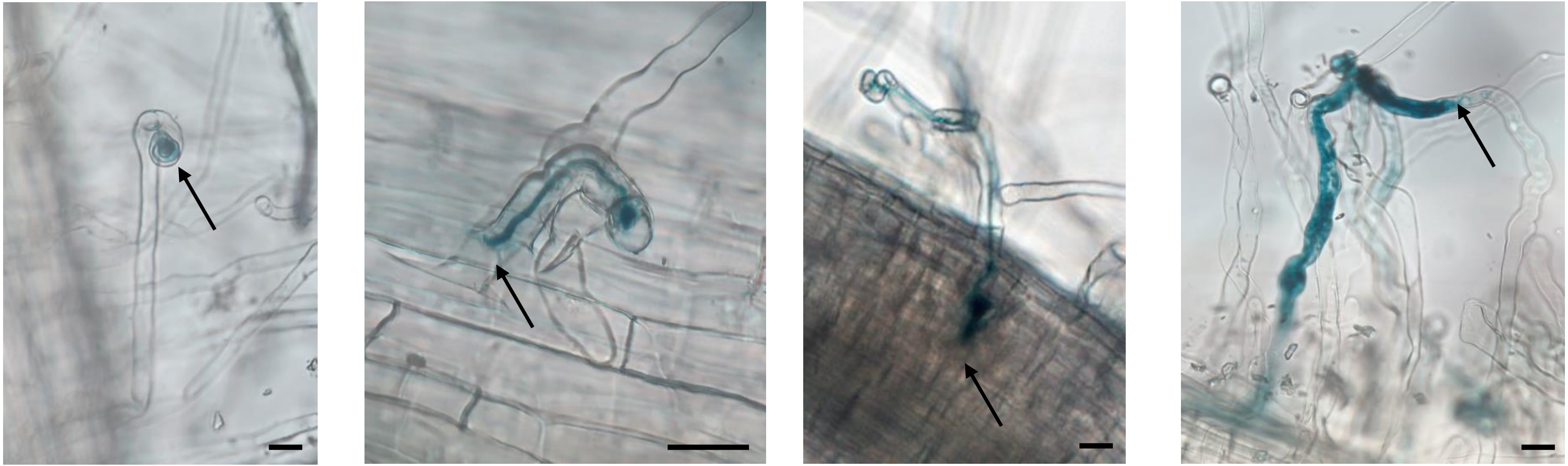

Microcolony Epidermal IT Cortical IT Abnormal IT

Supplementary Figure 3

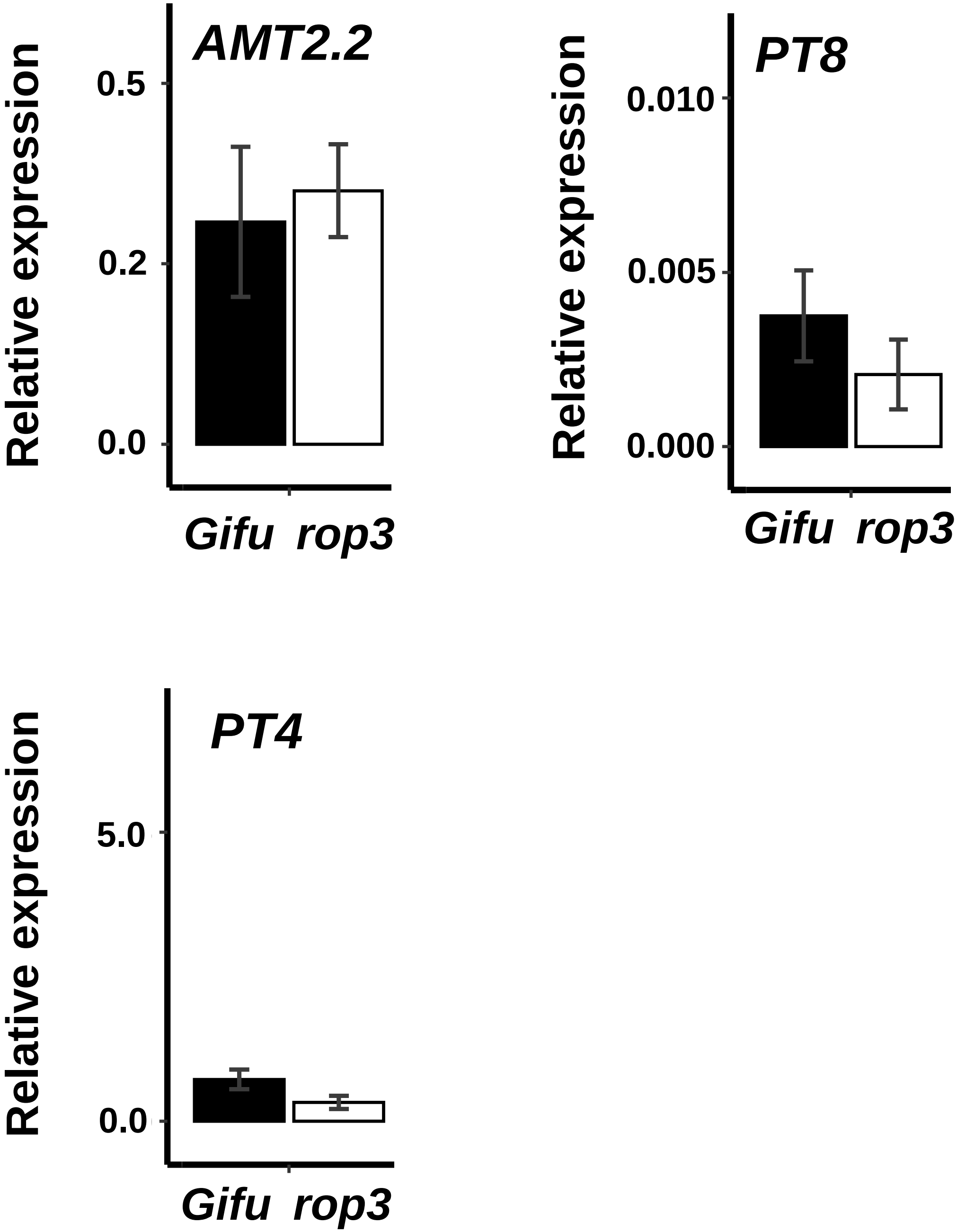

Supplementary Figure 4

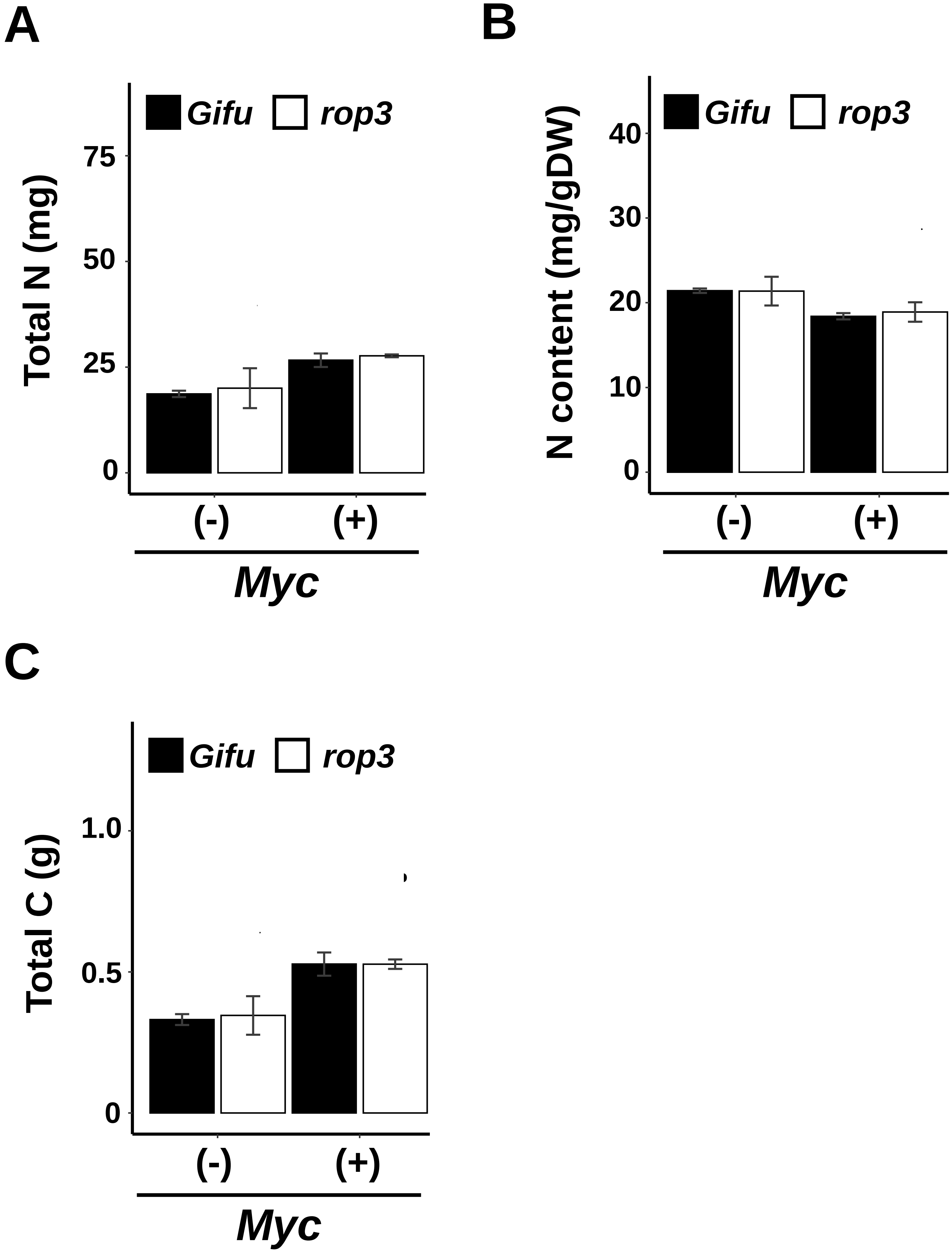

Supplementary Figure 5
